# Emerald eco-synthesis: harnessing oleander for green silver nanoparticle production and advancing photocatalytic MB degradation with TiO_2_&CuO nanocomposite

**DOI:** 10.1038/s41598-024-52454-0

**Published:** 2024-01-30

**Authors:** Amira M. Shawky, Rania Elshypany, Heba M. El Sharkawy, Mahmoud F. Mubarak, Hanaa Selim

**Affiliations:** 1 Sanitary and Environmental Institute (SEI), Housing and Building National Research Center (HBRC), Giza, 1770 Egypt; 2 Department of Analysis and Evaluation, Egyptian Petroleum Research Institute, Nasr City, Cairo 11727 Egypt; 3 Department of Petroleum Application, Core Lab Analysis Center, Egyptian Petroleum Research Institute, P.B. 11727, Nasr City, Cairo Egypt

**Keywords:** Climate sciences, Chemistry

## Abstract

The tertiary composite of TiO_2_/CuO @ Ag (TCA) were synthesized by the solid state method using different ratios of TiO_2_/CuO NCs and Ag NPs. The structural, morphological, and optical properties of nanocomposites were analyzed by scanning electron microscope, Transmission electron microscope, X-ray diffraction, Fourier transform infrared spectra, UV–Vis diffuse reflectance spectra (UV–Vis/DRS) and photoluminescence spectrophotometry. The results showed enhanced activity of TCA hybrid nano crystals in oxidizing MB in water under visible light irradiation compared to pure TiO_2_. The photocatalytic performance TCA samples increased with suitable Ag content. The results show that the photo degradation efficiency of the TiO_2_ compound improved from 13 to 85% in the presence of TiO_2_–CuO and to 98.87% in the presence of Ag containing TiO_2_–CuO, which is 7.6 times higher than that of TiO_2_. Optical characterization results show enhanced nanocomposite absorption in the visible region with long lifetimes between e/h+ at optimal TiO_2_–CuO/Ag (TCA_2_) ratio. Reusable experiments indicated that the prepared TCA NC photo catalysts were stable during MB photo degradation and had practical applications for environmental remediation.

## Introduction

Currently, the issue of energy crisis and environmental pollution is a topical and global issue, and solving these problems by different methods has been of particular interest^1,2^. The carcinogenic organic dyes are commonly used in the manufacture of various products as plastics, paper, textiles and paints. The wastewater discharged by these industries has serious impacts on the environment and on the health of humans and other organisms^3^. Most of these dyes are of synthetic origin and often include aromatic rings in their molecular structure, which are inert and cannot be degraded when released in wastewater without proper treatment^4,5^. Therefore, from the point of view of protecting human health and environmental resources, it is very urgent to remove such dyes from contaminated water^6^. Methylene blue (MB), the most commonly used basic dye, is thought to have a variety of uses in the printing and dyeing industry^7^. For this reason, several strategies have been described to remove MB from aqueous solutions, such as photocatalytic, biological and other conventional methods^8–10^. Among the methods utilised, photocatalysis is one that removes pollutants in an attractive and efficient approach^11,12^. One of the most promising methods to clean the environment is the photocatalytic removal of pollutants utilizing high performance semiconductor catalysts^13^. One of the most promising methods to clean the environment is the photocatalytic removal of pollutants via utilizing high performance semiconductor catalyst such as Titanium dioxide (TiO_2_)^14^. However, the applicability of TiO_2_ has been constrained due to poor photocatalytic activity in the visible area caused by the broad band gap (3.2 eV)^15^. In order to solve this issue and enhance the photocatalytic activity of TiO_2_, multiple approaches have been attempted, such as doping^16^, surface modification^17^ or a combination of semiconductor oxides^18^. Considering such modifications, visible light can be used to create electron-holes and extend their separation time, enhancing the photocatalytic activity^19^. Copper oxide (CuO ) nanoparticles, are among the metal oxides, which have a band gap energy of 2.1 eV^18^, have drawn a lot of attention because of their low cost, nontoxicity, good optical and catalytic capabilities, and superior activity in the visible spectrum^20,21^. In order to degrade Rhodamine B by UV–visible light, Ravishankar et al. used CuO/TiO_2_ nanocomposite as a photocatalyst. Under ideal circumstances, 98% of Rhodamine B dye was degraded in the presence of this nanocomposite. Bathla and colleagues reported the use of CuO/TiO_2_ nanocomposite to remove methyl orange dye when exposed to sunlight. They degraded that after 8 h of exposure to sunlight^22^. Bae et al. investigated the photocatalytic activity of hollow CuO/TiO_2_ nanospheres for the degradation of methylene blue. According to their findings, the photocatalytic performance of the nanospheres enhanced when exposed to visible light, and 90% of the methylene blue was destroyed^23^. Silver nanoparticles (SNP) have drawn a lot of interest because of their superior catalytic abilities during the dye oxidation process. SNP synthesis is often accomplished using a variety of physicochemical techniques. The majority of these methods have been demonstrated to be effective for the creation of SNP. However, they come with built-in drawbacks such the need for expensive chemicals, corrosive nature, and potential dangers to the environment. Utilizing an environmentally friendly strategy that involves organic SNP synthesis using plant-based extracts may be the solution to these negative consequences^24,25^. Numerous physical and chemical processes result in the production of nanoparticles that are hazardous or harmful for the environment. The biological approach and green chemistry are two common safer methods used by researchers to create nanomaterials^26^. Because it is so simple to manipulate, the green chemistry method has been widely used for the quick synthesis of AgNPs^27^. Researchers are adopting environmentally friendly processes to synthesise a variety of metallic nanoparticles since there is an increasing need for environmentally friendly nanoparticles. However, plant extract has been employed as a reducing agent to create nanoparticles that may be useful for the time being^26^. This study focusing on the effectiveness of prepared a novel silver nanoparticles by green chemistry from *Nerium oleander* as photocatalyst and enhanced its activity with nanocomposite TiO_2_@ CuO for MB photo-degradation in water by visible light irradiation.

## Experimental

### Materials

The chemicals used in this work are: Titanium (IV) isopropoxide (TIPO) [Ti(OCH(CH_3_)_2_)_4_], copper nitrate tri hydrate Cu(No_3_)_2_ 3H_2_O and silver nitrate AgNO_3_ are supplied by Sigma-Aldrich companies. Sodium hydroxide (NaOH) of purity (99%), Ethanol, methylene blue dye were purchased from Merck KGaA (Darmstadt, Germany) and Nerium oleander leaves. All solutions were made with fresh deionized water. All chemicals used in this work were of analytical grade and were used without further purification. The Study complies with local and national regulations and guidelines.

### Nanoparticles synthesis

#### Synthesis of TiO_2_ nanoparticles (T)

TiO_2_ NPs were prepared using a sol–gel method^5^. In a typical synthesis, an appropriate amount of Ti-isopropoxide precursor was mixed with deionized water and dissolved in 87.5 mL of ethanol, stirred at room temperature for 4 h, washed several times with deionized water, and dried at 90 °C. Finally, the obtained powder was calcined in air in a muffle furnace at 500 °C for 1 h to extract TiO_2_ NPs.

#### Synthesis of CuO nanoparticles (C)

CuO nanoparticles were prepared by a precipitation method^28^. A solution of Cu(No_3_)_2_ 0.3H_2_O (0.1 M) was dissolved in 100 mL of distilled water with continuous stirring. Add 0.1 M NaOH to the above solution until the pH reaches7. The color of the solution immediately changed from light blue to black and a large amount of black precipitate was formed. The precipitate was centrifuged and washed 3–4 times with deionized water. The resulting precipitate was air dried for 24 h. This CuO powder was used to characterize the material.

#### Synthesis of TiO_2_&CuO nancomposite (TC)

A TiO_2_/CuO nanocomposite (TC) was synthesized by a solid-state method^29^. TiO_2_/CuO composites were prepared using different weight ratios of T:C (0.1:0.08, 0.1:0.1, and 0.1:0.12 wt%) labeled as TC_1_, TC_2_, and TC_3_, then milled together, sonicated in a prope sonicator for 15 min. It was washed for several minutes, and then calcinated at 400 °C for 4 h.

#### Preparation of Ag NPs by green synthesis

##### Preparation of AgNO_3_ solution

0.5 g of AgNO_3_ was dissolved in distilled water (50 mL), then the solution was stirred at 60–70 °C for 15 min. until it is homogeneous. The solution is stored in a dark container to avoid oxidation.

##### Preparation Nerium oleander leaves extract

The oleander leaves were washed with water several times, sterilized with 50% ethanol to remove foreign substances such as dust, washed with distilled water several times, and cut into 25 cm pieces. An equal amount of gram pieces of oleander leaves was mixed with 100 ml of distilled water and boiled in a sterile flask at 80–90 °C for 5–10 min. until the water turned green color. The solution was filtered and stored in a refrigerator at 3 °C^30^.

##### Synthesis of silver nanoparticles (A)

For the synthesis of AgNPs, a certain amount of oleander extract (1 mL) was dropped into a suitable amount of AgNO_3_, and the aqueous solution was heated at 80 °C for 15 min. The resulting solution was pale yellow, indicating the formation of AgNPs.Hence; nerium oleander leaves extract was used as a bio-functional reducing material for the green synthesis of silver nanoparticles (Ag NPs)^31^.

#### Synthesis of TiO_2_&CuO @ Ag nancomposite (TCA)

A TiO_2_/CuO/Ag nanocomposite (TCA) was synthesized by a solid-state method. The optimal ratio of TC composite and Ag was established by using different weight ratios of TC:A (0.1:0.03, 0.1:0.05, and 0.1:0.06 wt%) are mentioned as TCA_1_, TCA_2_, and TCA_3_, and then milled together, sonicated for 15 min. Washed several times by distilled water and ethanol, and calcined at 400 °C for 4 h.

### Experimental techniques

The morphology of the prepared materials was examined with a scanning electron microscope (SEM) (JEOL) and a JEOL transmission electron microscope (TEM) associated with selected area electron diffraction (SAED). The phases of the prepared samples were examined by his X-ray diffraction (XRD) using a diffractometer (Panalytical XPERT PRO MPD). CuKα radiation (λ = 1.5418 Å) was used at 40 kV and 40 mA. Functional groups were identified in the wavenumber range 400–4000 cm^−1^ using a Fourier transform infrared (FT-IR) spectrometer model Spectrum One (Perkin Elmer, USA). Light reflectance was recorded using a UV–Vis spectrometer (Perkin Elmer Lambda 1050). Photoluminescence spectra were recorded on a Cary Eclipse fluorescence spectrophotometer.

### Photocatalytic activity study

The photocatalytic decomposition activity was examined using a photoreactor equipped with a 400 W halogen lamp as light source. The distance between the halogen lamp and the dye solution is 10 cm. Then, 0.025 g of photocatalyst was added to 30 ml of 20 ppm MB dye solution, and the solution was stirred in the dark for 30 min to reach adsorption–desorption equilibrium. Photolysis was started in 120 min and 5 ml of suspension was obtained in 30 min. The obtained suspension was analyzed by UV–Vis spectrophotometer at the MB solution's maximum absorption wavelength of 664 nm^32^.

## Results and discussion

**Figure 1:**
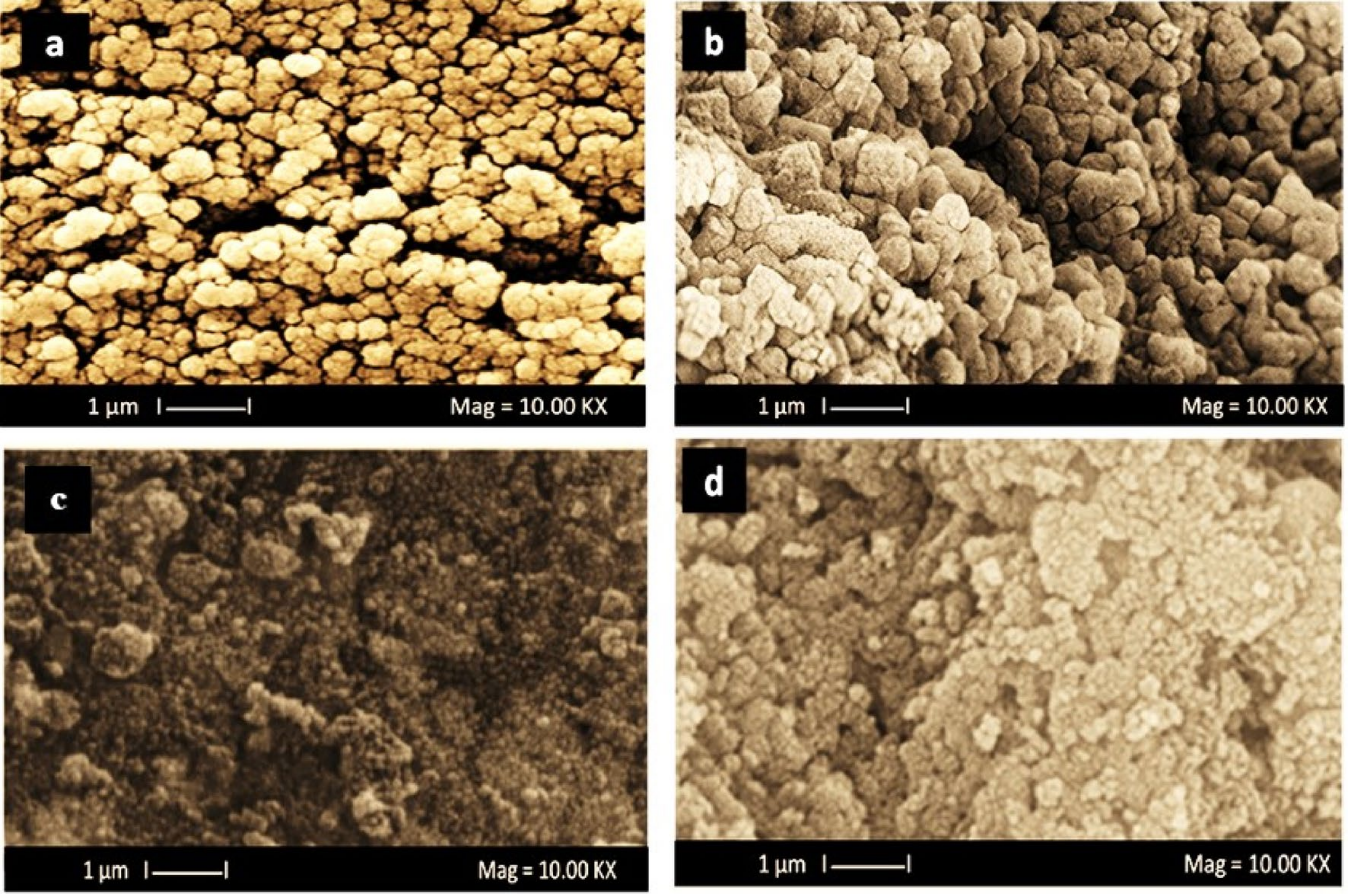
SEM images of the pure TiO_2_ (**a**), CuO (b), TC_1_ (**c**); and TCA_2_ (**d**) nanocomposites.

The SEM images of the most efficient photocatalyst of (0.05%) TiO_2_/CuO@Ag nanocomposite (TCA_2_), TC_1_ and pure TiO_2_, CuO nanoparticles are shown in Fig. 1. The SEM image of pure anatase TiO_2_ grains has rounded shape and form sponge-like aggregates as presented in Fig. 1a, and the global and uniform particles indicated in images of CuO and TC_1_ are coherent together as shown in Fig. 1b,c. There was also a higher tendency of agglomerations. In Fig. 1d indicates the SEM graph of net composite TiO_2_/CuO@ Ag, hence; the composite of TiO_2_ with CuO appears also spherical shapes and Ag nanoparticles with even shape and spherical nature, which aggregated on the surface of the composite. This evident the composite is successfully prepared.

**Figure 2:**
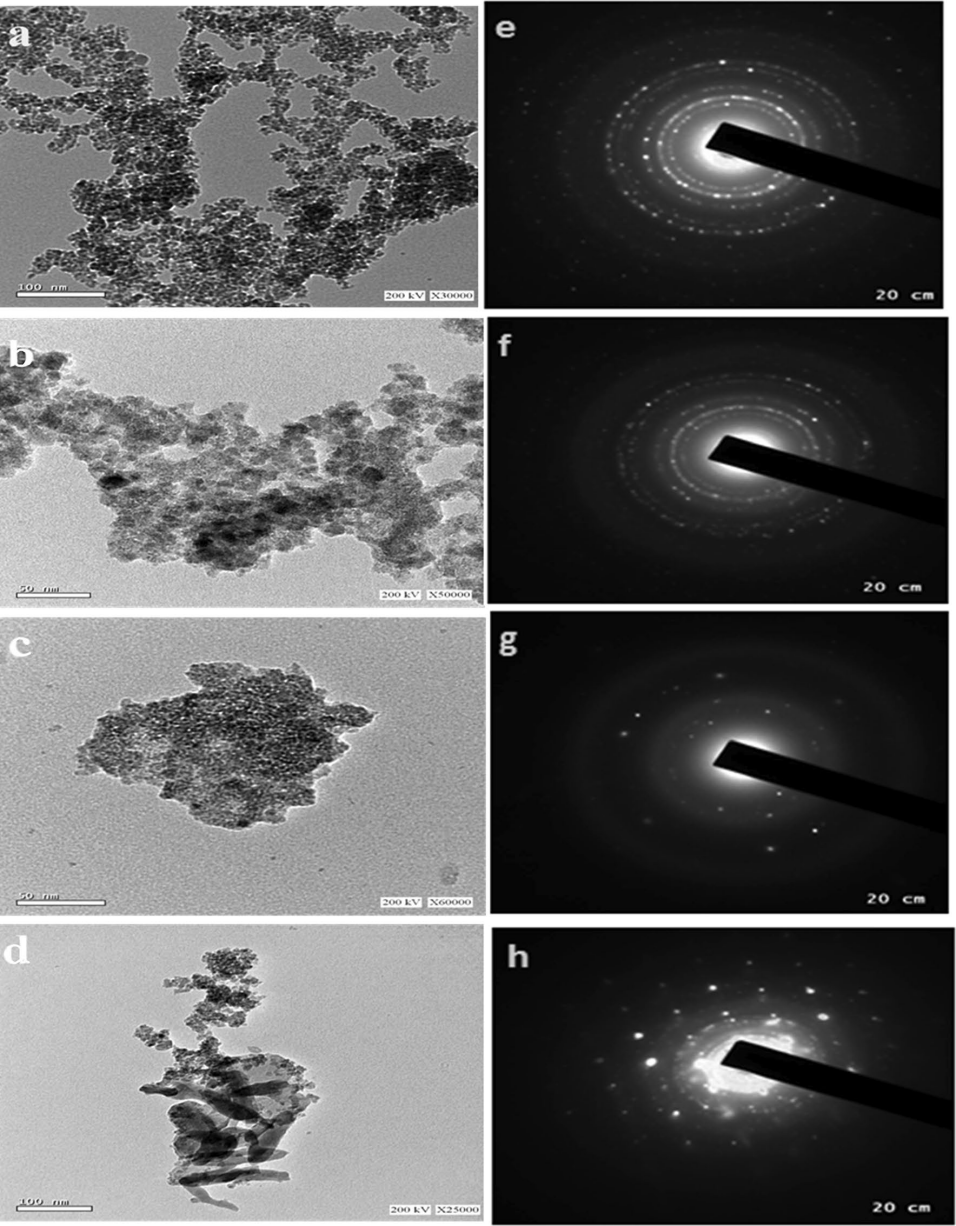
TEM images of the pure TiO_2_ (**a**), CuO (**b**) TC_1_ (**c**); and TCA_2_ (**d**) nanocomposites; and their SAED patterns (**e**–**h**), respectively.

The TEM micrographs of the pure and composite samples are represented in Fig. 2. The figure shows that nanoparticles have almost spherical shape corroborating the images of the SEM. Other essential results can be gotten from Fig. 2 is that the particle sizes of the TC are more aggregated than that found for pure one. While the TCA composite the sliver was appeared as Irregular sphere. Moreover, a series of bright diffraction rings were observed through the corresponding ring pattern of the selected-area electron diffraction (SAED), suggesting the polycrystalline nature of the prepared material, Fig. 2e–h.

**Figure 3:**
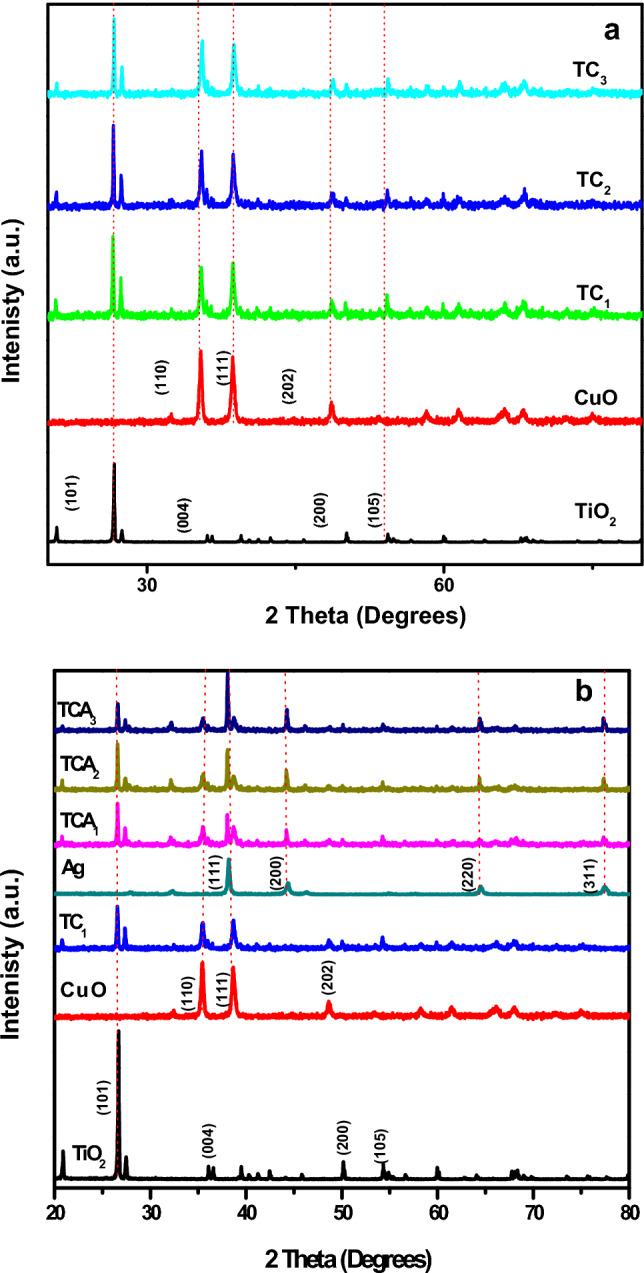
XRD pattern of TiO_2_ and CuO with (**a**) TC_1_, TC_2_ and TC_3_; and (**b**) TCA_1_, TCA_2_ and TCA_3_ nanocomposites.

The X-ray diffraction spectra was used to illustrate the purity, crystal structure and the phase formed for the as prepared nanocomposites. Figure 3 was displayed the X-ray diffraction spectra of bare samples of TiO_2,_ CuO, and Ag along with nanocomposites of TiO_2_/CuO and TiO_2_/CuO@Ag with different weight percentages of CuO and Ag, respectively. The diffraction peaks at 2θ of 26.69°, 36.13°, 50.05° and 54.40° corresponding to lattice planes (101), (004), (200) and (105), respectively for TiO_2_ anatase structure. Additionally, diffraction peaks characteristic to monoclinic CuO were noticed at 2θ of 35.44°, 38.81° and 48.70° representing to planes (110), (111) and (202), respectively. Upon adding CuO with various ratios of TiO_2_ the resultant nanocomposits (TC) contain mixed diffraction peaks of both CuO, TiO_2_ and the intensity of peaks of TiO_2_ was slightly decreased without the observation of any foreign peaks, conforming the successful formation of TC composites with high purity as shown in Fig. 3a. Furthermore, the main peaks of nano silver appeared at 2θ values of 38.02°, 44.37°, 64.32°, and 77.22° corresponding to planes of (111), (200), (220) and (311), respectively. Figure 3b revealed the Bragg reflections of silver after addition of Ag to TC composites with different ratios, new main peaks were detected approximately at 2θ of 43.58°, and 64.32°, and 77.22° which indexed well with the XRD patterns of Ag nanoparticles. It is noted that the diffraction peaks have some sharpness, demonstrating the great crystallinity of the prepared photocatalysts.

**Figure 4:**
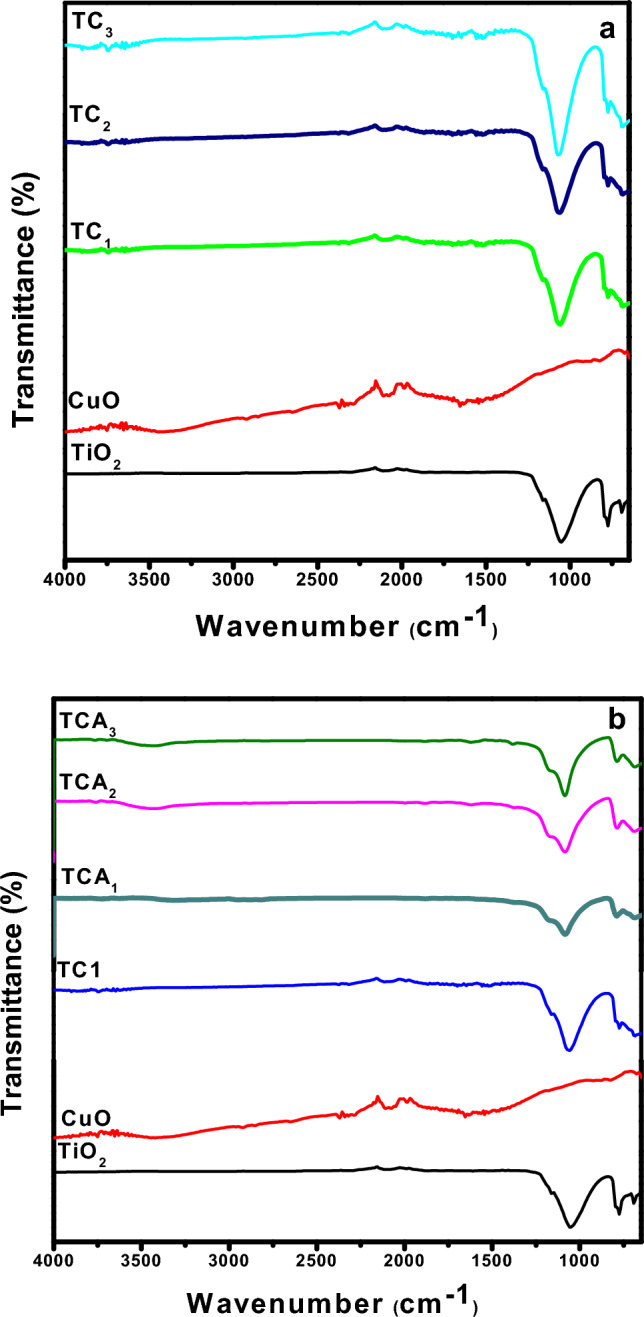
FT-IR Spectra of TiO_2_ and CuO with (**a**) TC_1_, TC_2_, and TC_3_; and (**b**) TCA_1_, TCA_2_ and TCA_3_ nanocomposites.

Figure 4 shows the FT-IR spectra of TiO_2_, CuO and TiO_2_/CuO @ Ag composite photocatalysts with mole ratios of Ag of 0%, 0.03%, 0.05%, and 0.06% for TC_1_, TCA_1_, TCA_2_ and TCA_3_ nanocomposites, respectively. The FT-IR spectrum of TiO_2_ shows strong absorption bands in the range of 400–700 cm^-1^. This band is assigned to the stretching vibration of the Ti–O–Ti bond and indicates the formation of TiO_2_ at 646 cm^−1^^33^. In Fig. 4a, the FT-IR spectrum of CuO shows peaks at 718.49, 848.53 and 960.67 cm^−1^ revealed the formation of CuO. A broad peak noticed at 3371.57 cm^−1^ attributed to O–H stretching of the moisture content^34^. For TC spectrum shows the presence of CuO oxide will give two characteristic low-intensity peaks of CuO vibrations in the 400–800 cm^−1^ region^35^. However, the spectrum of TiO_2_ will appear in the same region 400–800 cm^−1^, two high intensity peaks appeared for Ti–O vibrations^36^. It is easy to predict that the high-intensity characteristic peaks of TiO_2_ will overlap the low-intensity characteristic peaks of CuO in this wavenumber region. That is why there is no difference in characteristic peaks in the presence or absence of CuO oxide in the photocatalysts. Figure 4b revealed the FT-IR spectra of TiO_2_–CuO/Ag nanocomposites, which show all the characteristic vibrations of TiO_2_, CuO and silver, characteristic absorption peak at 1090.72,801.81, 690.40,504.43and 476.31 cm^−1^, hence; the peaks observed at 1090.72, and 960.67 cm^−1^ indicate the presence of silver nanoparticles^37^. Based on these results, a photocatalyst was prepared that did not contain any unnecessary foreign substances.

**Figure 5:**
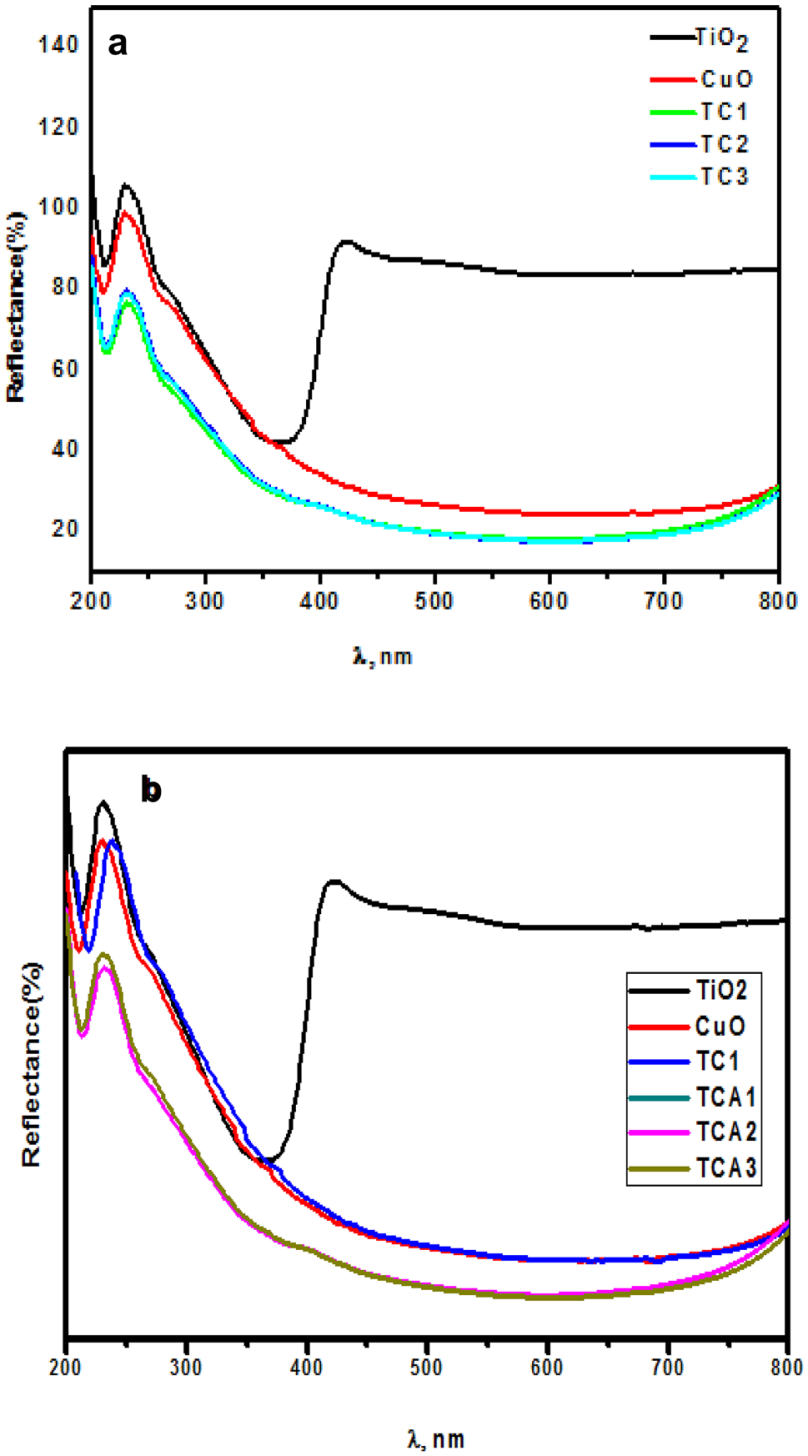
UV–Vis diffuse réflectance spectra of TiO_2_ and CuO with (**a**) TC_1_, TC_2_ and TC_3_; and (**b**) TCA_1_, TCA_2_ and TCA_3_ NCs.

The photocatalytic performance of a semiconductor significantly depends on its optical property, and thus, it is one of the important factors which should be studied. Figure 5a shows typical diffuse reflectance spectroscopy (DRS) curves for optical absorption behavior of pristine TiO_2_ and CuO and their composites TC_1_, TC_2_, and TC_3_ in the range of 200–800 nm. It was observed that the Pure TiO_2_ photocatalyst displayed almost the strongest absorption intensity at 422 nm compared with its counter parts. Furthermore, the pristine CuO nanocomposite showed relatively small absorption band edge around 270 nm that is compatible with the small band gap of CuO 1.3 eV. As for TC_1_, TC_2_ and TC_3_ NCs, It can be seen the low intensity of reflectance, indicating the highly absorbance of light in visible range based on the reduction of the band gap energy. It is proposed that the combination of n-type and p-type semiconductors produces an internal electric field, resulting in the production of a p-n hetero junction. The creation of this p-n hetero junction, as well as the band alignment between CuO and TiO_2_, considerably facilitates electron–hole separation and increases catalytic activity^38–40^. Next, the best performing photocatalyst, TC_1_ was modified with Ag nanoparticles as shown in Fig. 5b. Hence; Ag NPs modification of photocatalysts TCA_1,_ TCA_2_ and TCA_3_ endowed the TiO_2_/CuO @ Ag ternary composites the lowest reflectance, the optimum composite TCA_2_, as a result of the synergetic effect among components^41,42^. It has been demonstrated that lowering the composite's band gap energy improves the photocatalytic reaction by allowing the produced photocatalyst to absorb more photons and become more sensitive to visible light. The conjugation of two bands gap increased the stability between the e^−^/h^+^ pairs.

**Figure 6:**
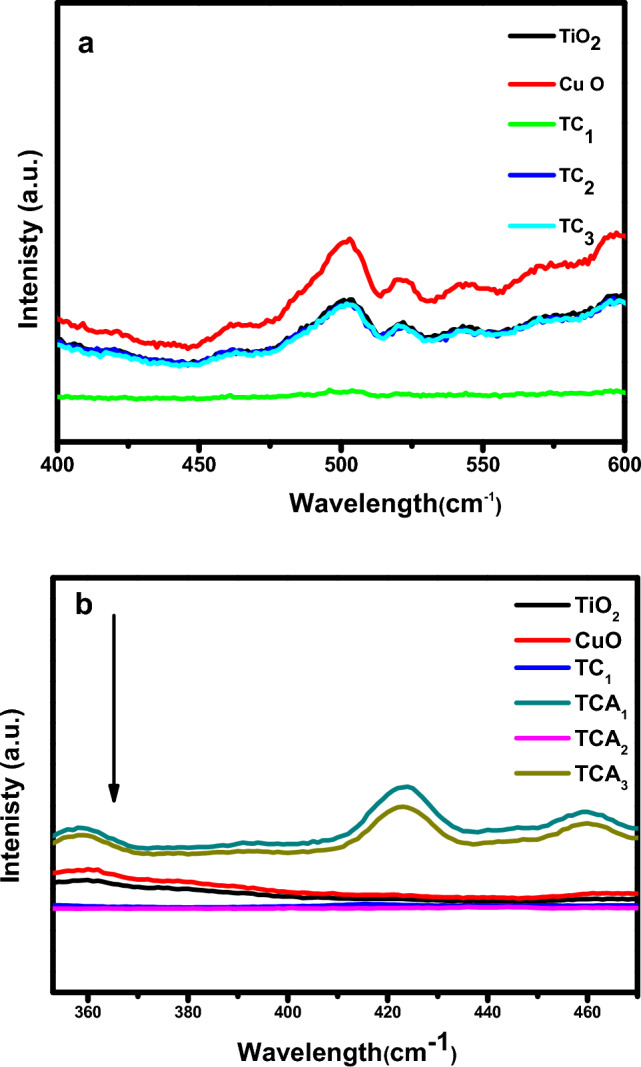
(**a**) Photoluminescence spectra of TiO_2_ and CuO with TC_1_, TC_2_, TC_3_; and (**b**) TCA_1_, TCA_2_ and TCA_3_ NCs.

The PL spectra demonstrate the behavior of photo generated charge carriers (e^−^, h^+^),where a low recombination rate of photogenerated electrons and holes means a good separation between (e^−^, h^+^), a low fluorescence intensity in PL spectra and consequently excellent photo degradation performance. Figure 6a,b displays the PL measurements of TiO_2,_ CuO, TC, and TCA nanocomposites^43^. The bare photocatalys, CuO, TiO_2_, and their nanocomposites (TC_1_, TC_2_, TC_3_) exhibte emission peaks around wavelength range of 470 to 550 cm^−1^, referring the recombination of photogenerated charge carriers in visible light region as shown in Fig. 6a. Moreover, a strong PL quenching is observed for TC_1_, TC_2_, and TC_3_ nanocomposites. TC_1_ photocatalyst displays the smallest PL intensity, indicating that the recombination between photo-electrons generated and holes is reduced^44^. Remarkably, Fig. 6b reveals the PL emission of TCA ternary composite, TiO_2_/CuO @ Ag NCs was further quenched after the addition of Ag nanoparticles to extremely low intensity, indicating improved carriers life time and effective electron hole separation. The p-n heterojunction of CuO/TiO_2_ align with the Schottky junction created with metallic Ag are responsible for facilitating charge separation and transfer through the interfaces^45^. Hence, TCA_2_ nonocomposite represents a lower recombination rate provides greater dye degradation efficiency for the photocatalyst, consistent with the photocatalytic results.

**Figure 7:**
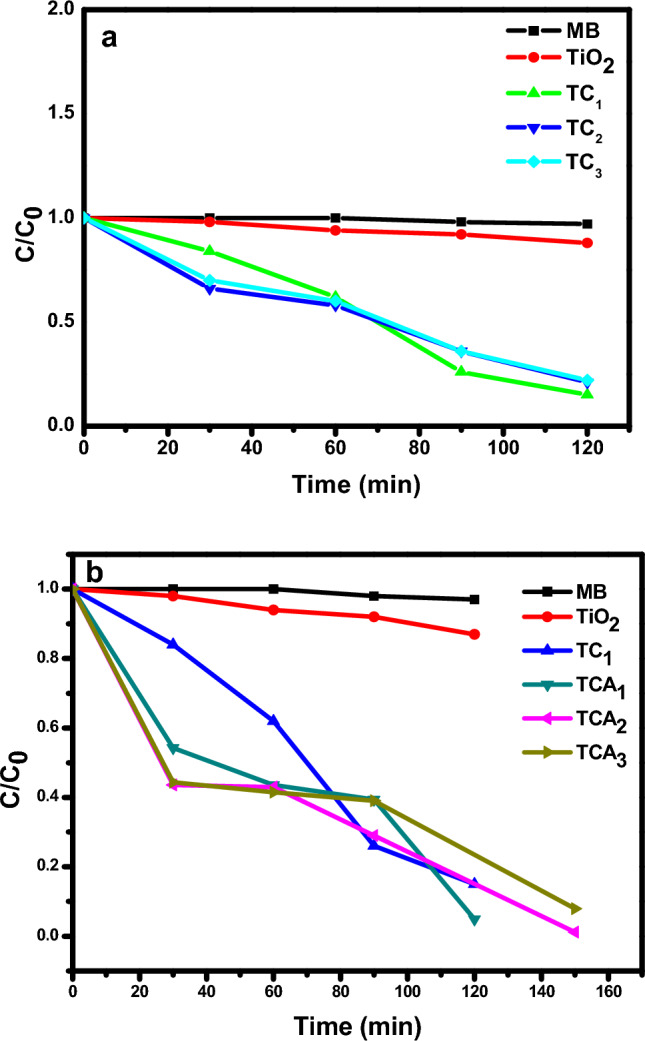
Photocatalytic degradation of MB by TiO_2_ (**a**) TC_1_, TC_2_, TC_3_; and (**b**) TC_1,_ TCA_1_, TCA_2_ and TCA_3_ NCs.

Using MB dye as a model pollutant, the photocatalytic activity of the produced TiO_2_, TC and TCA nanocomposites was examined as demonstrated in Fig. 7a,b. The photocatalytic efficiency of MB decomposition was calculated from the Eq. (1):

1 $${\text{D }}\% \, = \, \left( {{\text{C}}_{0} - {\text{C}}/{\text{C}}_{0} } \right)*{1}00$$

where C_0_ is the initial concentration and C is the remaining concentration of MB after the reaction. Initially, to reach adsorption/desorption equilibrium, the solution was held in the dark for 30 min prior to light irradiation. On the other hand, it is obvious that the photocatalytic performance of pure TiO_2_ was lower than that for their composites (TC) during the irradiation times of 0, 30, 60, 90, 120 and 150 min. This attributed to the large band gap of TiO_2,_ it suffer from high rate recombination between the photo induced charge carriers (e^−^, h^+^)^46^. Form Fig. 7a revealed the combination of TiO_2_ and CuO the photocatalytic activity increases to reach (85%) for TC_1_ photocatalyst compared to pure TiO_2_ (13%), TC_2_ (79%) and TC_3_ (78%). Furthermore, maximum degradation efficiency was obtained by inclusion of a suitable portion of Ag nanoparticles to TC_1_ in the composite TiO_2_/CuO @ Ag NCs with a degradation efficiency reach to (98.87%) for TCA_2_ as shown in Fig. 7b. The higher photo catalytic degradation efficiency of TCA_2_ to MB dye may be related to a suitable amount of Ag nanoparticles in the composite resulting in a lower band gap energy and sufficient PL characteristic. Additionally, the lower and higher amount of Ag nanoparticles in the ternary composite, TCA, was exhibited a negative effect on photodegradation performance as for TCA_1_ and TCA_3_ with photo degradation activity of (95.06%) and (92.07%), respectively. Where the lower Ag loading may be delay the transfer of photo generated carriers while the higher Ag loading may be hinder the light absorption.

**Figure 8:**
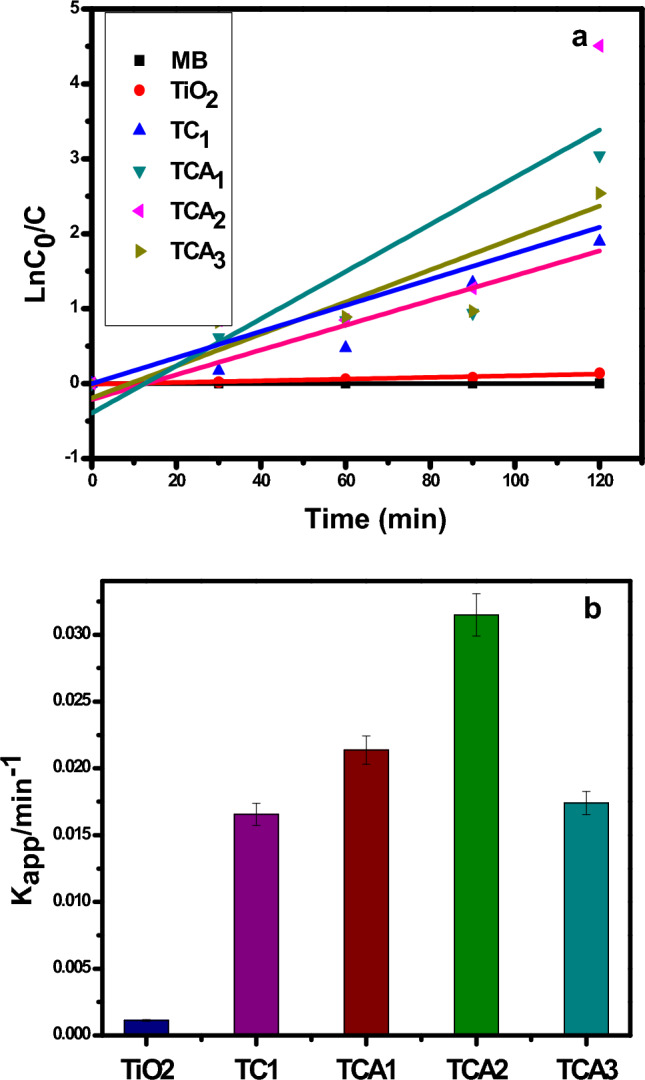
(**a**) Kinetics and; (**b**) apparent rate constants of MB degradation of by TiO_2_ with TC_1_, TCA_1_, TCA_2_ and TCA_3_ NCs.

The pseudo-first-order kinetic reaction of MB dye photodecomposition under visible light irradiation was explored in Fig. 8 and Table 1. The degradation kinetics of MB by the synthesized photo catalysts was calculated according to Eq. (2):

2 $${\text{ln}}\left( {{\text{C}}_{0} /{\text{C}}} \right) \, = {\text{ k}}_{{\text{a}}} *{\text{t}}$$

where C_0_ is the initial concentration (mg L^−1^), C is the reaction concentration of the MB solution at time (t), and k_a_ is the rate constant (min^−1^).

**Table 1:** The rate constant of nanocomposites (TiO_2_, TC_1_, TCA_1_, TCA_2_ and TCA_3_).

Sample	K_a_
MB	**0.0001**
TiO_2_	**0.00112**
TC_1_	**0.01655**
TCA_1_	**0.02137**
TCA_2_	**0.0315**
TCA_3_	**0.01741**

Significant values are in bold.

The linear relationship between the photodegradation time and dye concentration in the ln C_0_/Ct versus t time (t) plot implies that the degradation process was pseudo-first-order and, photocatalytic rate constant can obtained from it as shown in Fig. 8a. Further, the super photocatalytic performance of TCA_2_ is proofed from the photocatalytic rate constant value. It could be realized from Fig. 8b and Table 1 that the photocatalytic rate constant of nano composites (TC_1_, TCA_1_, TCA_2_ and TCA_3_) was more than that of pristine bare TiO_2_ taking the order of TCA_2_ (0.0315 min^−1^) > TCA_1_(0.02137 min^−1^) > TCA_3_ (0.01741 min^−1^) > TC_1_ (0.01655 min^−1^) > T (0.00112 min^−1^) > MB (0.0001min^−1^). Additionally, adding Ag nanoparticles to TC1 photocatalyst was resulted in increased k_a_ value. The greatest rate constant (0.0315 min^−1^) belongs to the TCA_2_ photo catalyst. Which is consistent with the results of photocatalytic degradation, signifying that the catalyst has good features toward MB degrading activity under visible light.

Table 2 provides a comparison of the photocatalytic efficiency of various catalysts in the degradation of a dye called Methylene Blue (MB) with respect to a synthesized nano-composite called TiO_2_/CuO @ Ag. The table provides information on the photocatalyst, dye concentration, catalyst dose, preparation method, degradation percentage, and the corresponding reference for each entry.

**Table 2:** Comparison of photocatalytic efficiency of relevant catalysis with respect to our synthesized TiO_2_/CuO @ Ag nano-composite.

Photocatalyst	Dye type	Dye concentration (ppm)	Catalyst dose (g/L)	Preparation methode	Degradation (%)	Ref.
Ag@Cu_2_O CuO/TiO_2_	MB	20	0.5	Sol–gel technique	83.00	^42^
CuO-TiO_2_	MB	20	1	Green synthesis usingC.benghalensis plant extracts	33.00	^47^
Ag@CuO	MB	20	2	Apolyol-mediated refluxing method	94.43	^42^
Ag/TiO_2_/CuFe_2_O_4_	MB	5	1	Hydrothermal method	85,00	^48^
TiO_2_@CoFe_3_O_4_	MB	100	1.0	Co-preciptation method	91,00	^5^
Pt/ZnO-MWCNT	MB	100	0.4	sol–gel method	74,00	^47^
Ag@CuO/TiO_**2**_	MB	20	0.5	Green synthesis using nerium oleander leaves extract	98.87	This work

The Ag@Cu_2_O–CuO/TiO_2_ catalyst was prepared using the sol–gel technique. It was tested against MB dye at a concentration of 20 ppm, with a catalyst dose of 0.5 g/L. The degradation percentage achieved was 83.00%^42^. Additionally, CuO-TiO_2_ catalyst was synthesized using a green synthesis method involving *C. benghalensis* plant extracts. The MB dye concentration used was 20 ppm, and the catalyst dose was 1 g/L. The degradation achieved was 33.00%^47^. Also, the catalyst of Ag@CuO that prepared using an apolyol-mediated refluxing method, was tested against MB dye at a concentration of 20 ppm with a catalyst dose of 2 g/L. It achieved a degradation percentage of 94.43%^42^. Furthermore, the hydrothermal method was used to synthesize of Ag/TiO_2_/CuFe_2_O_4_ photocatalyst and then tested against MB dye at a concentration of 5 ppm, with a catalyst dose of 1 g/L. The catalyst exhibited degradation efficiency of 85.00%^48^. The degradation efficiency of 91% was achieved via TiO_2_@CoFe_3_O_4_ nanocomposite against MB dye at a concentration of 100 ppm, with a catalyst dose of 1.0 g/L. TiO_2_@CoFe_3_O_4_ catalyst was synthesized using the co-precipitation method^5^. The sol–gel method was further used to prepare Pt/ZnO-MWCNT catalyst and tested against MB dye at a concentration of 100 ppm, with a catalyst dose of 0.4 g/L, achieving 74%^47^. Finally, Ag@CuO/TiO_2_ nano-composite that synthesized for the current work. The MB dye concentration used was 20 ppm, and the catalyst dose was 0.83 g/L. It was prepared using a green synthesis method involving nerium oleander leaves extract. The degradation achieved by this composite was 98.87%. In summary, the table compares the photocatalytic efficiency of relevant catalysts, including the TiO_2_/CuO @ Ag nano-composite synthesized in the current work. It demonstrates that the TiO_2_/CuO @ Ag composite showed the highest degradation percentage (98.87%) among all the tested catalysts for the degradation of MB dye at a concentration of 20 ppm and a catalyst dose of 0.5 g/L.

**Figure 9:**
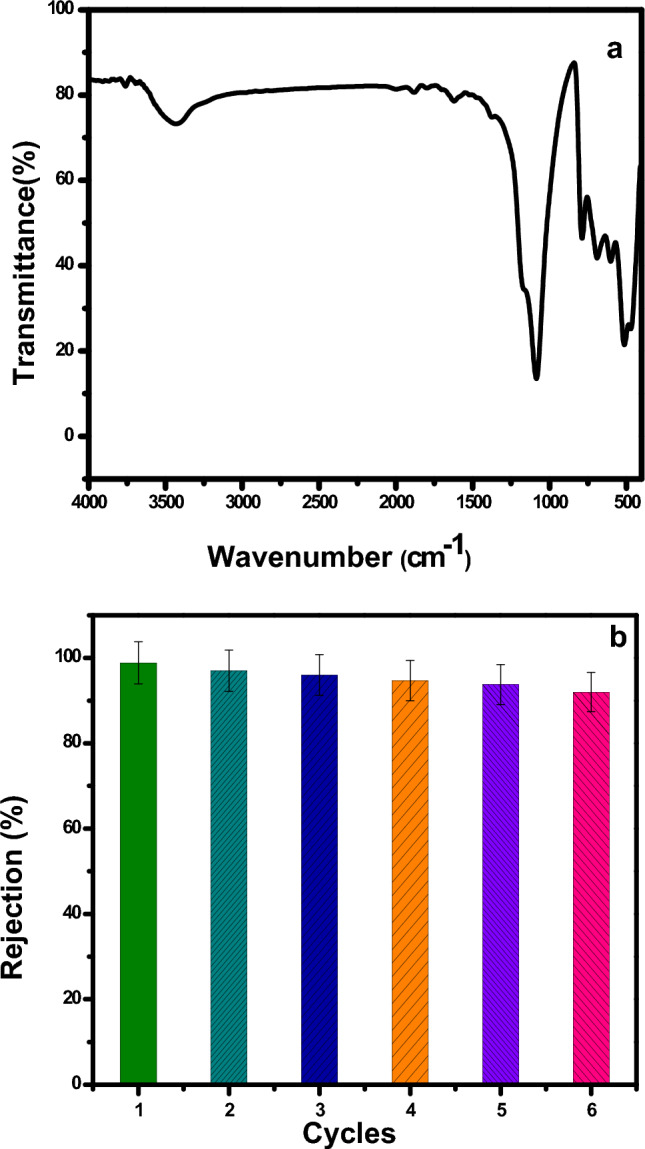
(**a**) FT-IR spectra and; (**b**) Reusability of TCA_2_ after photocatalytic degradation for MB.

Reusability experiments were conducted using FT-IR after 120 min of photocatalysis, as can be seen in Fig. 9a. There was no change in the peak position, and the peaks exactly matched those of the FT-IR catalysis from before the photocatalytic degradation reaction. These findings explain how the produced catalyst can keep its stability and catalytic effectiveness even after numerous repeat usage. As demonstrated in Fig. 9b, the optimized photocatalyst TCA_2_ for MB degrades at a rate that can be recycled, and after six cycles of usage, its photo degradation activity was found to slightly decline, showing strong stability. The interaction of TiO_2_@CuO and Ag, which can immobilize silver nanoparticle active sites in photocatalysis, is responsible for stability feature of TCA NCs.

**Figure 10:**
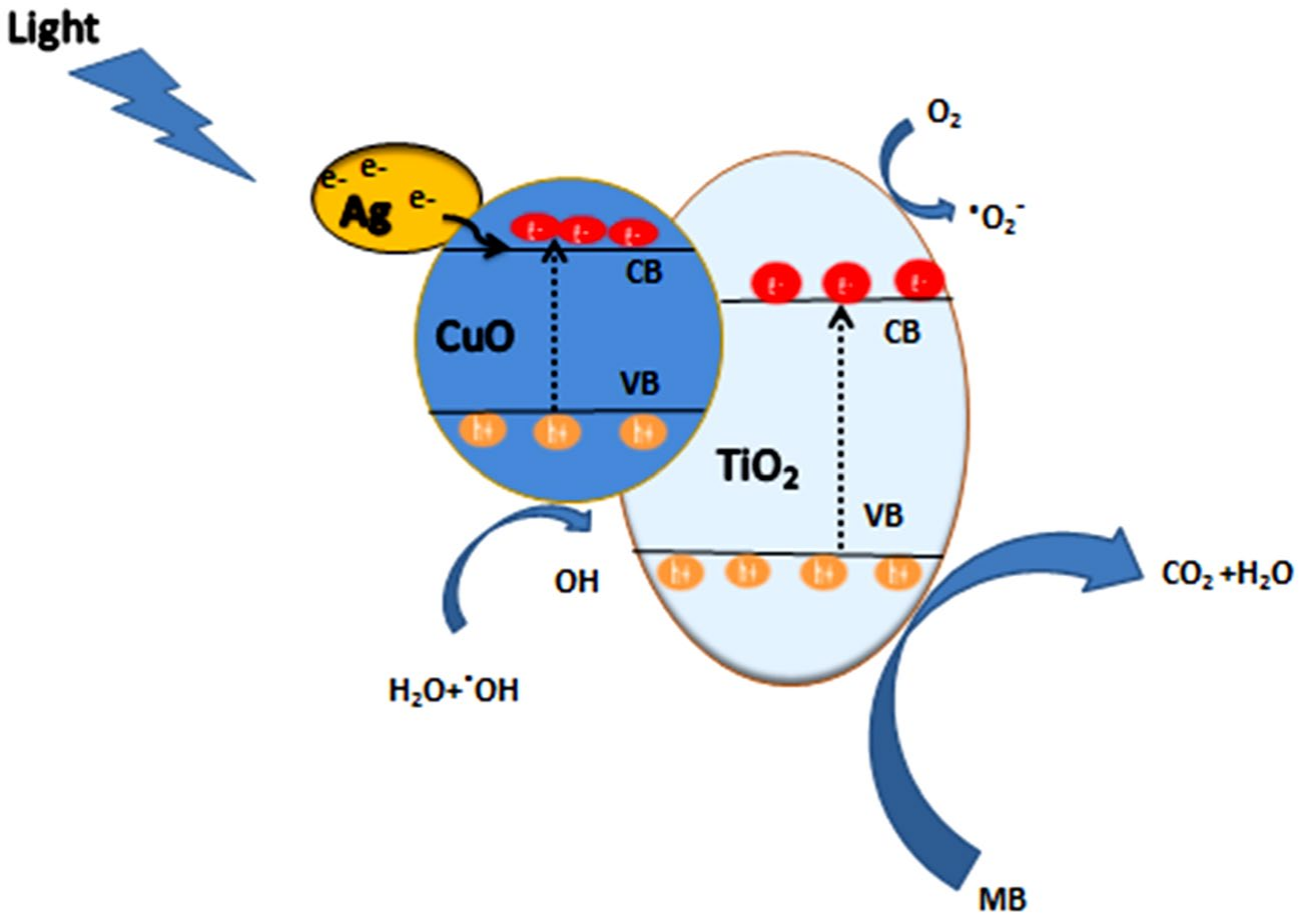
Schematic diagram of the mechanism for the photo catalytic of MB by TCANCs under visible light irradiation.

The photocatalytic dye degradation process includes light absorption, generation of electron–hole pairs, and redox reactions with the dye adsorbed on the surface of the photocatalyst, upon exposing the photocatalysts to adequate light as shown in Fig. 10. which during the degradation process, the charge species such as OH^-^, h^+^, and e^-^ act as oxidizing and reducing agents, and OH^.^, O_2_^.^, and HO_2_^.^ radicals and holes (h^+^) are intermediates that react spontaneously on the neighboring ions, and degrading the dye compounds to environmental friendly CO_2_ and H_2_O as shown at the following Equations.

3 $${\text{TCA }} + {\text{ h}}\nu \, \to {\text{ h}}^{ + } + {\text{ e}} -$$

4 $${\text{TCA}} + {\text{ O}}_{{2}} \to {\text{ O}}_{{2}}^{. - }$$

5 $${\text{O}}_{{2}}^{. - } + {\text{ 2H}}_{{2}} {\text{O }} \to {\text{ 4OH}}^{.}$$

6 $${\text{h}}^{ + } + {\text{ H}}_{{2}} {\text{O }} \to {\text{ OH}}^{.} + {\text{ H}}^{ + }$$

7 $${\text{H}}_{{2}} {\text{O }} + {\text{ H}}^{ + } + {\text{ O}}_{{2}}^{. - } \to {\text{ H}}_{{2}} {\text{O}}_{{2}} + {\text{ OH}}^{.}$$

8 $${\text{H}}_{{2}} {\text{O}}_{{2}} + {\text{ e}}^{ - } \to {\text{ OH}}^{ - } + {\text{ OH}}^{.}$$

9 $${\text{h}}^{ + } + {\text{ OH}}^{ - } \to {\text{ OH}}^{.}$$

10 $${\text{OH}}^{.} + {\text{ organic pollutant }} \to {\text{ degradation to CO}}_{{2}} + {\text{ H}}_{{2}} {\text{O}}$$

The developed photocatalyst, TiO_2_/CuO@Ag has a p-n heterojunction formed as a result of the combination between p-type (CuO) and n-type (TiO_2_) semiconductors. Furthermore, the conduction band (CB) level of TiO_2_ is higher than the CB level of CuO. Leading to the diffusion of holes from p-type to n-type region and electrons from n-type to p-type via the heterojunctions or to the attached Ag nanoparticles. As a result, it makes the separation of photogenerated electrons and holes easier along with the influence of Ag nanoparticles' surface plasma resonance (SPR) that enhances the collection of visible light harvesting. Resulting in enhanced photocatalytic efficiency^39,49–51^.

## Conclusion

In conclusion, this study successfully synthesized a green-synthesized TiO_2_/CuO@Ag nanocomposite using Nerium oleander leaves extract. The TCA nanocomposite exhibited superior photocatalytic activity for the degradation of MB under visible light. The presence of Ag in the nanocomposite significantly enhanced its photocatalytic efficiency, achieving a degradation percentage of 98.87% for MB. The TCA nanocomposite demonstrated stability and reusability, making it a promising material for environmental remediation applications. This research contributes to the development of sustainable and efficient photocatalytic materials for water treatment and pollution control. Further investigations can explore optimization and scalability of the TCA nanocomposite for broader environmental applications.

## Data Availability

All data underlying the results are available as part of the article and no additional source date are required.
